# Severe Hypercalcemia Due to Primary Hyperparathyroidism and Heterozygous Pathogenic Variant of CYP24A1

**DOI:** 10.1210/jcemcr/luad071

**Published:** 2023-08-09

**Authors:** Jannel Liu, Peter Angelos, Maan Barhoum, Rajesh Jain

**Affiliations:** Department of Endocrinology, Diabetes, and Metabolism, University of Chicago Medicine, Chicago, IL 60637, USA; Department of Endocrine Surgery, University of Chicago Medicine, Chicago, IL 60637, USA; Advanced Diabetes & Endocrine Center, Libertyville, IL 60048, USA; Department of Endocrinology, Diabetes, and Metabolism, University of Chicago Medicine, Chicago, IL 60637, USA

**Keywords:** hypercalcemia, CYP24A1, vitamin D, hyperparathyroidism, parafibromin

## Abstract

Pathogenic variants of CYP24A1 are associated with hypercalcemia due to disruptions in the ability of 24-hydroxylase to break down 1,25-dihydroxyvitamin D (1,25-DHVD). A case involving a heterozygous pathogenic variant of CYP24A1 and primary hyperparathyroidism leading to severe hypercalcemia has not been previously reported. A 23-year-old woman presented with fatigue and was found to be hypercalcemic at 13.8 mg/dL [reference range, 8.4-10.2 pg/mL]. Her parathyroid hormone (PTH) was 62 pg/mL [reference range, 19-88 pg/mL] and 1,25-DHVD was elevated to 242.7 pg/mL [reference range, 18-72 pg/mL]. Other laboratory workup was unrevealing. She had a bone scan, whole body CT scan, and thyroid ultrasound that were normal. Her 25-hydroxy-vitamin D to 24,25-dihydroxy-vitamin D ratio was elevated at 25.18 (normal, < 25). Because of concern for primary hyperparathyroidism, she was referred to an endocrine surgeon and underwent a parathyroidectomy with the removal of a 3.5-gram adenoma. Pathology showed a parafibromin-deficient parathyroid neoplasm. Genetic testing demonstrated a heterozygous pathogenic variant in CYP24A1. Three weeks after surgery, PTH was 14 pg/mL (1.48 pmol/L), calcium and 1,25-DHVD normalized. In summary, we report a case where a patient with severe symptomatic hypercalcemia was found to have primary hyperparathyroidism exacerbated by an underlying heterozygous pathogenic variant in CYP24A1.

## Introduction

Hypercalcemia is a common clinical problem that can present with a wide range of symptoms and etiologies, with primary hyperparathyroidism being one of the leading causes of hypercalcemia [1]. The typical pattern of primary hyperparathyroidism is elevated calcium and parathyroid hormone (PTH) levels—in general, the severity of hypercalcemia is related to the severity of hyperparathyroidism. Here, we present a novel case of severe hypercalcemia with an inappropriately normal PTH level that was found to be multifactorial, involving primary hyperparathyroidism and a heterozygous pathogenic variant of CYP24A1.

## Case Presentation

A 23-year-old female with a past medical history of depression and anxiety but otherwise no medical conditions presented with severe fatigue and was found to be hypercalcemic to 13.8 mg/dL ([3.45 mmol/L]; reference range, 8.4-10.2 pg/mL [2.1-2.54 mmol/L]) with a PTH of 62 pg/mL ([6.57 pmol/L]; reference range, 19-88 pg/mL [19-88 ng/L]). She did not take any excessive dairy products, antacids, calcium, or vitamin D supplements, and she had no personal history of nephrolithiasis or fractures. She had no known family history of hypercalcemia, fractures, or nephrolithiasis. She was on no medications besides an oral contraceptive pill and escitalopram 20 mg daily. She had a calcium level of 9.8 mg/dL (2.45 mmol/L) 14 months prior to presentation. At her initial presentation, she was admitted to the hospital and received 4 liters of intravenous fluid and was discharged with a calcium of 11.7 mg/dL. She was re-admitted 2 weeks later with a calcium of 13.2 mg/dL (3.30 mmol/L). She was evaluated by nephrology and received intravenous fluids and intravenous pamidronate 30 mg. She was discharged with a calcium of 10.5 mg/dL (2.63 mmol/L). One month later, she was found to have a calcium of 12.7 mg/dL (3.17 mmol/L). As an outpatient, she received intravenous pamidronate 60 mg, and cinacalcet was titrated up to 90 mg twice daily over several weeks. However, she continued to develop hypercalcemia ranging from 11.5 to 12.3 mg/dL (2.88-3.08 mmol/L) and symptoms including weakness, fatigue, nausea, and decreased ability to focus. She required 2 doses of zoledronic acid 4 mg due to persistent symptomatic hypercalcemia over the subsequent 4 months.

## Diagnostic Assessment

An extensive laboratory evaluation for her hypercalcemia was performed during her clinical course (Table 1). Her labs showed a 25-hydroxyvitamin D (25-OHD) of 28.2 ng/mL ([70.4 nmol/L]; reference range, 30-100 ng/mL [74.88-249.60 nmol/L]), and given the unclear clinical picture and concern of overlying secondary hyperparathyroidism, she was started on vitamin D3 2000 international units (IU) daily.

**Table 1.** Laboratory evaluation of hypercalcemia showing the patient's results with reference ranges

Lab	Patient's results	Normal range
1,25-dihydroxyvitamin D	242.7 pg/mL (582.48 pmol/L)	18-72 pg/mL (43.20-172.80 pmol/L)
25-hydroxyvitamin D	28.2 ng/mL (70.4 nmol/L)	30-100 ng/mL (74.88-249.60 nmol/L)
24-hour urine calcium	150 mg/24 hour (3.75 mmol/24 hour)	100-300 mg/24 hour (2.5-7.5 mmol/24 hour)
ACE	32 U/L	<40 U/L (<666.68 nkat/L)
Albumin	4.0 g/dL (40 g/L)	3.4-5.4 g/dL (34-54 g/L)
BSALP	32 U/L (0.53 ukat/L)	10.5-44.8 U/L (0.18-0.75 ukat/L)
Calcitonin	2.0 pg/mL (0.58 pmol/L)	<10 pg/mL (<2.92 pmol/L)
Creatinine	0.72 mg/dL (353.6 umol/L)	0.59-1.04 mg/dL (52.16-91.94 umol/L)
Ferritin	43 ng/mL (96.62 pmol/L)	11-307 ng/mL (24.72-689.86 pmol/L)
Free T4	1.0 ng/dL (12.87 pmol/L)	0.8-1.5 ng/dL (10.30-19.31 pmol/L)
Magnesium	2.2 mg/dL (0.9 mmol/L)	1.7-2.2 mg/dL (0.7-0.9 mmol/L)
Metanephrines (plasma)	0.23 nmol/L (0.042 ng/mL)	<0.5 nmol/L (<0.091 ng/mL)
Phosphorus	2.1 mg/dL (0.68 mmol/L)	2.8-4.5 mg/dL (0.9 mmol/L-1.45 mmol/L)
PTH	62 pg/mL (62 ng/L)	19-88 pg/mL (19-88 ng/L)
PTHrP	<2.0 pmol/L (<2.0 pg/mL)	<2.5 pmol/L (<2.5 pg/mL)
Quantiferon-gold	Negative	
SPEP	Normal	
TSH	0.991 mIU/L (0.991 mIU/L)	0.35-5.0 mIU/L (0.35-5.0 mIU/L)

Abbreviations: ACE, angiotensin converting enzyme; BSALP, bone-specific alkaline phosphatase; PTH, parathyroid hormone; PTHrP, parathyroid hormone related protein; SPEP, serum protein electrophoresis; T4, thyroxine; TSH, thyroid stimulating hormone.

She underwent a thyroid ultrasound, Technetium-99m Sestamibi with single photon emission computed tomography (SPECT), and computed tomography (CT) scan of the neck, which were all read as normal. She was referred to hematology/oncology due to concerns for hypercalcemia of malignancy. The patient underwent a bone scan and CT scan of the chest, abdomen, and pelvis, which were normal.

**Figure 1.**
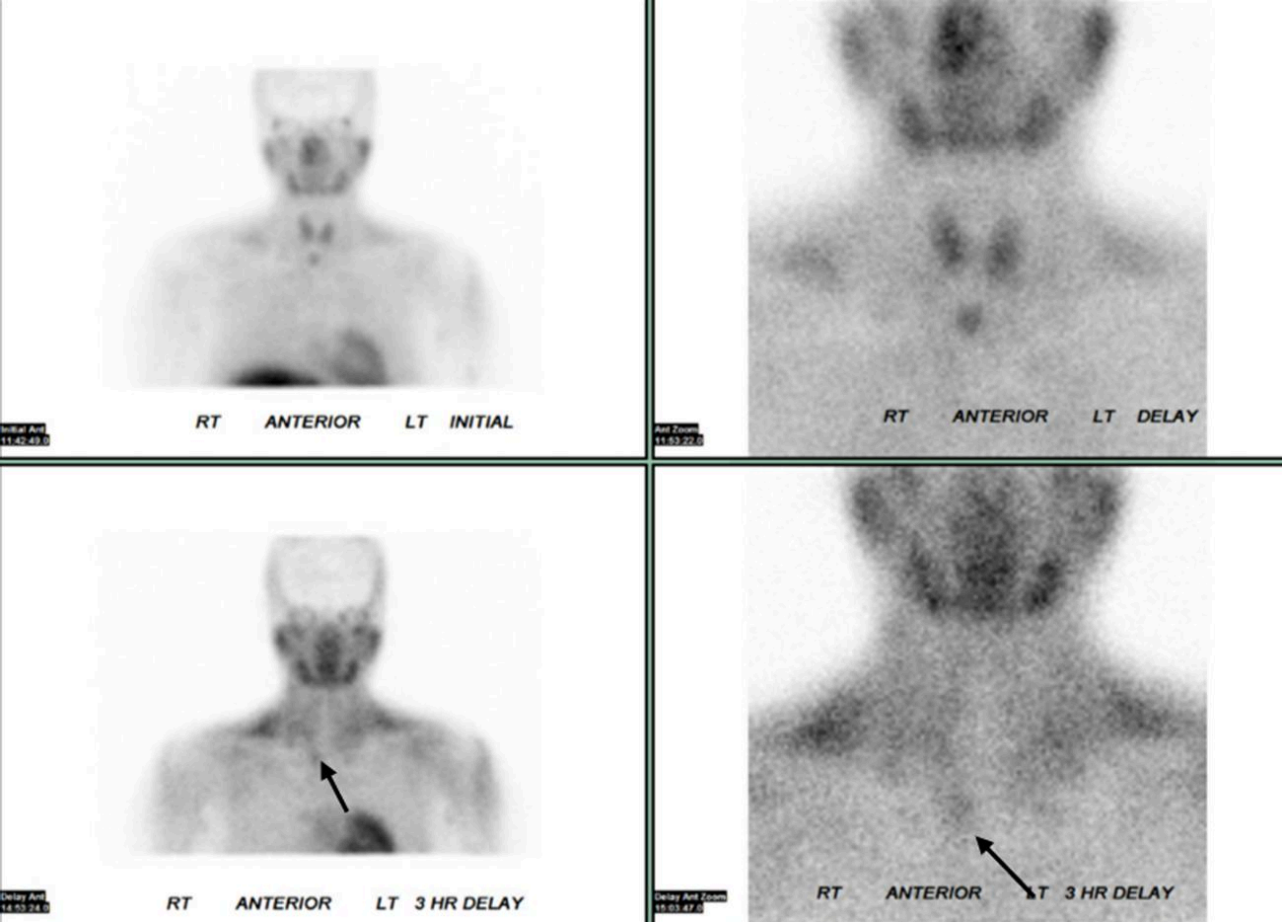
Technetium-99m Sestamibi scintigraphy showed persistent increased uptake (arrows) below the right lobe of the thyroid gland during the delayed phase.

Due to the lack of clarity on the diagnosis, she was referred to an academic metabolic bone disease center. There was concern for multiple etiologies of her hypercalcemia given the severe hypercalcemia, elevated 1,25-dihydroxyvitamin D (1,25-DHVD), and inappropriately normal PTH. Vitamin D supplements were stopped. A 25-hydroxyvitamin D (25-OHD) to 24,25-dihydroxyvitamin D (24,25-OHD) ratio was sent to Mayo Laboratories, which returned elevated at 25.18 (normal, < 25). Ratios over 25 can be seen in patients with pathogenic variants of CYP24A1. She was referred to an endocrine surgeon, who felt that her Technetium-99m Sestamibi scan showed persistently increased uptake below the right gland of the thyroid on delayed images, consistent with a parathyroid adenoma (Fig. 1).

## Treatment

During surgery, a 3.5-gram parathyroid adenoma was removed, with other glands appearing of normal size and consistency. Intraoperative PTH levels dropped from 117 pg/mL pre-incision to 36 pg/mL (36 ng/L) after 5 minutes and 23 pg/mL (23 ng/L) at 15 minutes. Postoperatively, the patient was prescribed calcium supplements, but not calcitriol.

## Outcome and Follow-up

Subsequent pathology showed a parafibromin-deficient parathyroid neoplasm. At this point, genetic testing was sent for CYP24A1, CDC73, AP2S1, CASR, CDKN1B, GNA11, MEN1, and RET (the latter 7 being part of a commercial hyperparathyroidism panel). The patient's CYP24A1 returned positive for a novel heterozygous pathogenic variant (p.Pro392Argfs*9, causing a premature stop codon), with all other genetic tests being negative. She had a low PTH level of 14 pg/mL (1.48 pmol/L) but normal albumin-adjusted calcium level 9.6 mg/dL (2.4 mmol/L). Her 1,25-DHVD levels normalized to 58 pg/mL ([39.2 pmol/L]; reference range, 18-72 pg/mL [43.20-172.80 pmol/L]). She was weaned off calcium supplements, advised to avoid vitamin D supplements without regular monitoring, and to be cautious about prolonged sun exposure. Genetic counseling was recommended.

## Discussion

Here, to our knowledge, we report the first case of severe hypercalcemia from primary hyperparathyroidism and CYP24A1 heterozygous pathogenic variant. Our case demonstrates how CYP24A1 pathogenic variants may alter the presentation of primary hyperparathyroidism, leading to severe hypercalcemia even with only mild elevations in PTH.

The CYP24A1 is a mitochondrial gene responsible for making the enzyme 24-hydroxylase. This enzyme catalyzes 1,25-DHVD and 25-OHD to their inactive forms. Biallelic pathogenic variants in this gene were found to be the cause of idiopathic infantile hypercalcemia, a disorder first discovered in the 1950s [2]. Subsequently, patients with biallelic and monoallelic pathogenic variants of CYP24A1 were reported to have elevated 1,25-DHVD and impaired 24-hydroxylase–mediated degradation [1]. Biallelic pathogenic variants in these genes led to hypercalcemia and clinical complications such as nephrolithiasis [3]. In comparison, monoallelic variations have been reported to have more mild clinical presentations [4, 5]. Small studies of patients with heterozygous CYP24A1 pathogenic variants have suggested a higher prevalence of hypercalciuria and risk of nephrolithiasis, although many patients had normal biochemistries [5]. Even in our patient, after her parathyroidectomy, her calcium and 1,25-DHVD levels normalized. Normal biochemical profiles are commonly found in patients with heterozygous pathogenic variants of CYP24A1. In a case series and review, 15 out of 22 subjects with heterozygous CYP24A1 pathogenic variants had normal calcium and 1,25 DHVD levels [5]. Additionally, despite having severe hypercalcemia, our patient had normal 24-hour urine calcium. The aforementioned review also demonstrated that 19 out of 24 patients with heterozygous pathogenic variants of CYP24A1 did not have elevated urinary calcium [5].

Vitamin D deficiency is commonly found and treated in patients with primary hyperparathyroidism, and studies suggest patients with vitamin D deficiency have more severe biochemical disease and lower bone density [6]. However, vitamin D replacement has been reported to exacerbate hypercalcemia in patients with homozygous or compound heterozygous pathogenic variants of CYP24A1. Other factors, including pregnancy or sunlight, have also been reported to worsen hypercalcemia in patients with pathogenic variants of CYP24A1 [7, 8]. Patients with heterozygous CYP24A1 pathogenic variants should take care to avoid excessive vitamin D supplementation and prolonged sun exposure to minimize the risk of precipitating hypercalcemia.

Patients with hypercalcemia secondary to CYP24A1 pathogenic variants typically show an appropriately low PTH as it is a PTH-independent pathway. Using the current understanding of CYP24A1 and its relation to vitamin D metabolism, the ratio of 25-OHD to 24,25-OHD was developed as a screening test for CYP24A1 deficiency. Both 25-OHD and 1,25-DHVD are hydroxylated by 24-hydroxylase which is encoded by CYP24A1. When this enzyme loses its function, the ratio of 25-OHD increases in relation to 24,25-OHD. Ratios greater than 80 suggest a biallelic CYP24A1 pathogenic variant. Ratios between 25 and 80 can be seen in patients with low vitamin D or heterozygous CYP24A1 pathogenic variants, as seen in our patient. Ratios less than 25 were normal but may also be observed in heterozygous carriers [9]. The test is run at Mayo Laboratories but can be sent through common commercially available laboratories, such as Quest Diagnostics.

The pathology in this patient's case showed a parafibromin-deficient tumor. Parafibromin protein is a tumor suppressor that is encoded by CDC73. Pathogenic variants in this gene are associated with hyperparathyroid jaw tumor syndrome, parathyroid cancer, and other head and neck, gastric, lung, colorectal, and ovarian malignancies [10]. Patients with parafibromin-deficient tumors should undergo genetic screening for CDC73 pathogenic variants. However, in this patient, no germline pathogenic variant was present for CDC73. Parafibromin proteins are known to be involved in the transcription in the nucleus and cell growth in the cytoplasm, while CYP24A1 proteins are involved in vitamin D metabolism in the mitochondria [3, 10]. There is currently no evidence suggesting a link between parafibromin and CYP24A1 proteins. We believe that in this case, the patient had 2 separate and unrelated pathologic processes that led to severe hypercalcemia.

In summary, we report a case in which a young woman with severe symptomatic hypercalcemia was found to have primary hyperparathyroidism that was exacerbated by an underlying heterozygous pathogenic variant in CYP24A1.

## Learning Points

- Underlying pathogenic variants of CYP24A1 can may alter the presentation of primary hyperparathyroidism and other common presentations of hypercalcemia.
- The 25-OHD to 24,25 OHD ratio can be used to screen for CYP24A1 deficiency.
- Patients with CYP24A1 pathogenic variants are predisposed to hypercalcemia when exposed to precipitating factors.
- Patients with CYP24A1 pathogenic variants should avoid excessive vitamin D supplementation and prolonged sun exposure to minimize risk of hypercalcemia.

## Contributors

All authors made individual contributions to authorship. P.A., M.B., and R.J. were involved in the diagnosis and management of this patient. P.A. was responsible for the patient's surgery. J.L., P.A., M.B., and R.J. were involved with manuscript submission. All authors reviewed and approved the final draft.

## Data Availability

Data sharing is not applicable to this article as no datasets were generated or analyzed during the current study.

## Abbreviations

1,25-DHVD: 1,25-dihydroxyvitamin D
24,25-OHD: 24,25-dihydroxyvitamin D
25-OHD: 25-hydroxyvitamin D
CT: computed tomography
PTH: parathyroid hormone
