# Correction to “Severe Hypercalcemia Due to Primary Hyperparathyroidism and Heterozygous Pathogenic Variant of CYP24A1”

**DOI:** 10.1210/jcemcr/luad125

**Published:** 2023-10-13

In the above-named article by Liu J, Angelos P, Barhoum M, and Jain R (*J Clin Endo Metab Case Reports*. 2023; 1(4); doi: 10.1210/jcemcr/luad071), there was an error in the title:

The gene *CYP24A1* was not italicized in the title of the article as originally published. The title should appear as “Severe Hypercalcemia Due to Primary Hyperparathyroidism and Heterozygous Pathogenic Variant of *CYP24A1*”.

Several instances of the gene in the body of the article have also been updated to be italicized.

The article has been corrected online.

doi: 10.1210/jcemcr/luad071

